# Multiple Porous Synthetic Bone Graft Comprising EngineeredMicro-Channel for Drug Carrier and Bone Regeneration

**DOI:** 10.3390/ma14185320

**Published:** 2021-09-15

**Authors:** Chun-Sik Bae, Seung-Hyun Kim, Taeho Ahn, Yeonji Kim, Se-Eun Kim, Seong-Soo Kang, Jae-Sung Kwon, Kwang-Mahn Kim, Sahng-Gyoon Kim, Daniel Oh

**Affiliations:** 1 College of Veterinary Medicine, Chonnam National University, Gwangju 61186, Korea; csbae210@chonnam.ac.kr (C.-S.B.); leicia@naver.com (S.-H.K.); thahn@jun.ac.kr (T.A.); ksevet@gmail.com (S.-E.K.); vetkang@chonnam.ac.kr (S.-S.K.); 2 OsteoGene Bio, 75 Oak Street, Norwood, NJ 07648, USA; yeonji27@gmail.com; 3 College of Dentistry, Yonsei University, Seoul 03722, Korea; jkwon@yuhs.ac (J.-S.K.); kmkim@yuhs.ac (K.-M.K.); 4 College of Dental Medicine, Columbia University, New York, NY 10032, USA; drmartinkim@gmail.com

**Keywords:** synthetic bone graft, micro-channel, multiple pores, drug carrier, bone regeneration

## Abstract

Due to high demand but limited supply, there has been an increase in the need to replace autologous bone grafts with alternatives that fulfill osteogenic requirements. In this study, two different types of bone grafts were tested for their drug carrying abilities along with their osteogenic properties. Two different types of alendronate-loaded bone grafts, Bio-Oss (bovine bone graft) and InRoad (biphasic synthetic bone graft) were observed to see how different concentrations of alendronate would affect the sustained release to enhance osteogenesis. In this study, defected ovariectomize-induced osteoporotic rat calvarias were observed for 28 days with three different concentrations of alendronate (0 mg, 1 mg, 5 mg) for both Bio-Oss and InRoad. A higher concentration (5 mg) allowed for a more controlled and sustained release throughout the 28-day comparison to those of lower concentrations (0 mg, 1 mg). When comparing Bio-Oss and InRoad through histology and Micro-CT, InRoad showed higher enhancement in osteogenesis. Through this study, it was observed that alendronate not only brings out robust osteogenesis with InRoad bone grafts, but also enhances bone regeneration in an alendronate-concentration-dependent manner. The combination of higher concentration of alendronate and multiple porous bone graft containing internal micro-channel structure of InRoad resulted in higher osteogenesis with a sustained release of alendronate.

## 1. Introduction

Performed in over two million patients worldwide, the bone grafting procedure is the second most prevalent tissue transplantation [1]. Currently, autologous bone grafts are the preferred method over other procedures because it contains important properties—osteoconduction, osteoinduction, and osteogenesis, etc.—to ensure a successful bone transplantation [2]. Unfortunately, complications such as inferior healing and limited supply compared to the high demand of autologous bone grafts require different alternatives that would suffice osteogenic properties mentioned above [3,4,5].

Biphasic calcium bone grafts are rising alternatives to stimulate cell growth within the porous structure, whether it is through the seeding or migrating of cells from nearby tissues. In an ideal design, synthetic bone grafts need to be equipped for cell migration, proliferation, attachment, and differentiation. In addition, synthetic bone grafts can qualify as drug carriers that can supply to the site of transplantation. This drug delivery system would bypass the need for cells to be locally seeded in order to increase tissue repair and regeneration [6,7,8,9,10,11]. Moreover, many other studies focused on bone regeneration utilizing induced osteoporotic animal models were performed in subcritical size defects [12,13,14]. Even with the established proof of bone regeneration models, critical size defects need to be promoted for the confirmation of translational feasibility and regenerative capacity of synthetic bone graft materials [15].

Previous studies have shown that the wicking property of highly porous, multi-level configurational hydroxyapatite bone void filler (HA-BVF) granules can organically stimulate healing cascade without the addition of exogenous factors or cells [15]. The result concluded that the new bone formation of HA-BVF outperformed that of Bio-Oss, one of the most popular clinically used bone grafting materials in dentistry [15,16]. Also, the uniformly packed granules used in the study are known to support cell migration and extracellular matrix (ECM) growth in the vacant spots within the granules [17]. However, many studies have raised concerns that scaffolds and granules or any type of implants are open to the possibilities of becoming infected, requiring drug release from the implant materials [18]. Therefore, in this study, the dual function of HA-BVF for drug carrying and its bone regeneration ability was explored with the addition of alendronate.

Osteoporosis is defined by decreasing bone mass, deteriorating bone tissue, and degrading bone microarchitecture [19]. Although osteoporosis is widespread, the advancement for the utilization of synthetic bone graft to resolve deteriorating bone density and bone mass has been lacking greatly [20,21,22,23]. Bisphosphonates such as alendronate, risedronate, zoledronate, etc., are a class of drugs that prevent the loss of bone density, commonly used to treat osteoporosis and similar diseases. Based on clinical studies, there are pros and cons among the drugs. No significant differences were observed through the studies. Hence, in this study, we decided to use alendronate as a model drug to test preclinical feasibility and capability of InRoad for bone regeneration together with drug carrier. Furthermore, alendronate is the preferred choice of bisphosphate because of their efficacy, administration method, cost efficiency, and the availability of long-term safety data [24,25,26,27,28]. The effect of alendronate was proven through numerous and extensive studies to significantly impact osteogenesis by decreasing bone turnover through bone resorption inhibition [29,30]. In addition, the specific type of granule used for this study has micro-channels and submicron holes that would advance drug loading and releasing behavior [31].

Hypothesizing that the addition of alendronate will enhance bone regeneration using the localized drug carrying ability, HA-BVF named InRoad synthetic bone graft was observed and compared with Bio-Oss, a popularly used allograft. In this study, in vivo calvaria bone regeneration was observed using ovariectomize-induced osteoporotic rat models. Calvaria critical size (5 mm in diameter) defect models were created to test the hypothesis. For a deeper understanding of bone regeneration impact associated with Aln, three different concentrations of Aln (0 mg, 1 mg, and 5 mg) were examined for 4, 8, and 12 weeks using both Bio-Oss (bovine bone graft) and InRoad (biphasic synthetic bone graft; 90 ± 5% hydroxyapatite (HA) and 10 ± 5% β-tricalcium phosphate (β-TCP)). Through the comparison between popular allograft (Bio-Oss) and synthetic HA-BVF (InRoad), the osteogenic properties from InRoad compared to that of Bio-Oss was also observed. All the results were analyzed through a scanning electron microscope, microtomographic, histomorphometric, and histological evaluation.

## 2. Materials and Methods

**Figure 1:**
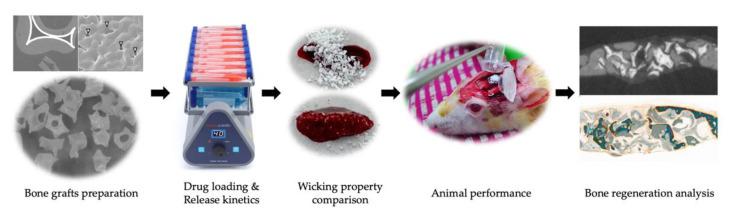
Graphical abstract of the study.

This study was carried out to evaluate the feasibility and capability for bone regeneration together with drug carrier in comparison between commercial xenograft (Bio-Oss) and newly developed synthetic calcium phosphate bone graft (InRoad) (Figure 1).

### 2.1. Preparation of Alendronate (Aln) Loaded Bone Grafts

InRoad bone grafts ranging 0.3–1.0 mm in diameter were fabricated following the procedure, identical to the previous fabrication [15]. Briefly, 200 to 400 nm sized biphasic powder was mixed with mixing solution with a 1.7–1.8 powder/solution ratio. The mixing solution was made of 1.0 wt% poly(vinyl alcohol) (Sigma-Aldrich, Milwaukee, WI, USA), 0.1 wt% carboxymethyl cellulose (Sigma-Aldrich, Milwaukee, WI, USA) as binders, and 1.0 wt% ammonium polyacrylate (R.T. Vanderbilt Company, Norwalk, CT, USA) as an anionic dispersant. The prepared biphasic paste was coated onto a polyurethane sponge template and sintered at 1230 °C for 3 h. Then, the sintered scaffold blocks were granulated using a mortar and pestle and sieved with correlating experimental size. The obtained granule should have a porous cancellous-bonelike structure containing internal micro-channel. And 0.25–1 mm range in diameter Bio-Oss was used in this study as a comparison. In order to prepare the Aln-loaded bone grafts, 1 mg and 5 mg of Aln was dissolved in 0.1 M MES buffer (pH 5.6). Bone grafts were then immersed in solution and underwent gentle shaking to react for 24 h. Aln-loaded bone grafts were collected after shaking, washed with distilled water, and vacuum-dried for one day.

### 2.2. Characterization of Aln-Loaded Bone Grafts (BGs)

The surface morphologies, overall and internal structures of bone grafts were investigated using scanning electron microscopy (SEM, JEOL 5700, Tokyo, Japan) and micro-computed tomography (micro-CT: SKYSCAN 1727, Billerica, MA, USA) was performed. Elemental analysis of Aln (1 mg)/BGs and Aln (5 mg)/BGs was assessed using X-ray diffraction.

### 2.3. Release Kinetic of Aln from Bone Grafts

For the in vitro kinetic evaluation of Aln release from Aln (1 mg)/InRoad or Bio-Oss and Aln (5 mg)/InRoad or Bio-Oss, each sample was immersed in 1 mL of PBS buffer (pH 7.4) with gentle shaking (100 rpm) at 37 °C. At predetermined time periods of 1, 3, 5, and 10 h, and 1, 3, 5, 7, 14, 21, and 28 days, the supernatants of the specimens were collected and replaced with an equal volume of fresh PBS solution. To record the absorbance, the wavelength was set at 293 nm with a complex of Aln and standard iron (III) chloride solution, using a Flash Multimode Reader (Varioskan™, Thermo Scientific, Waltham, MA, USA).

### 2.4. Animals

All procedures were authorized under the Institutional Animal Care and Use Committee of Chonnam National University (CNU IACUC-YB-2019-30). Animal care protocols were under abidance with the Guidelines for Animal Experiments of Chonnam National University. General anesthesia was used for all surgical procedures and tramadol was used for postoperative analgesic care in effort to lessen animal distress. A total of 90 Sprague–Dawley rats (Female, 11 weeks old, 251.9 ± 10.15 g; Samtaco, Osan, Korea) were operated and observed. All 90 rats were distributed appropriately in a random manner into 6 different groups (n = 5). The bone regeneration process of each group was observed and assessed at 4, 8, and 12 weeks following the bone grafts implantation. For each observation date, a control group was set up for Bio-Oss (Geistlich, Wolhusen, Switzerland; bovine bone graft small particles 0.25–1.0 mm) and experimental group for InRoad (OsteoGene Tech, Norwood, NJ, USA; dental synthetic bone graft small particles 0.3–1.0 mm) [15]. Then, the rats were further divided into three groups with different concentrations of Aln, leading to total of six different groups:

- Group I: Aln (0 mg)/Bio-Oss;
- Group II: Aln (1 mg)/Bio-Oss;
- Group III: Aln (5 mg)/Bio-Oss
- Group IV: Aln (0 mg)/InRoad;
- Group V: Aln (1 mg)/InRoad;
- Group VI: Aln (5 mg)/InRoad.

### 2.5. Ovariectomize (Ovx) Operation and Defect Surgery

**Figure 2:**
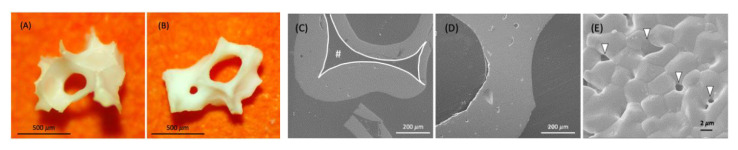
Digital and SEM comparison images between InRoad (**A**,**C**,**E**) and Bio-Oss (**B**,**D**). Both graft types similar under stereo microscope (**A**,**B**). Cross-section image under SEM showed distinguishable difference in internal structure; InRoad showed an embedded micro-channel (**C**) and Bio-Oss did not (**D**). Micro-sized holes (arrowhead) were observed on the InRoad surface (**E**).

Preoperatively, the rats were first fasted for 12 h then weighed. Rats were also under subcutaneous treatments with atropine (0.1 mg/kg; Jeil Pharmaceutical, Daegu, Korea) and enrofloxacine (2.5 mg/kg; Bayerkorea, Seoul, Korea). Using intraperitoneal injection, rats were placed under anesthesia with a mixture of xylazine (10 mg/kg; Bayerkorea, Seoul, Korea) and ketamine (40 mg/kg; Yuhan Co., Seoul, Korea). Under a sterile environment, two separate flank incisions were made through the epidermis and muscle. Once the ovaries became visible, ovariectomy was carried out by first placing a hemostat to hold the uterine horns after pulling the ovary gently. Then, a 4–0 silk ligature was placed below the hemostat. The ovary was extracted, and the uterus was placed back to the abdomen. Operated abdominal muscle layer was sutured back with absorbable 4–0 suture (Surgisorb, Samyang Co., Seoul, Korea). Epidermis was sutured back with non-absorbable 3–0 suture (Silk, Ailee Co., Busan, Korea) and treated subcutaneously with 3 mL of normal warmed saline. After 8 weeks post operation, bone graft was performed using the exact same anesthesia protocol to that of ovariectomy. Then, to prepare for the skin incision, the rat head was shaved and disinfected. After the epidermis was cut, an L-shaped incision was made to the exposed periosteum. The incision area was separated from the skull with blunt scraping. Then, using a trepan bur (5 mm in diameter), a circular critical size bone defect was made. The void of the defected area was filled with either Bio-Oss or InRoad (Figure 2) with appropriate Aln concentration. After filling the void, the periosteum was sutured back with absorbable 4–0 suture. Epidermis was sutured back with non-absorbable 3–0 suture and treated subcutaneously with tramadol (10 mg/kg) at 12, 24, and 36 h for continued postoperative analgesia.

### 2.6. Analysis of Bone Formation

Calvaria harvest was performed at 4, 8, and 12 weeks post bone graft implantation. The manually selected region of interest (ROI) comprised of the defected area. Harvest calvarias were stored in 10% buffered formalin for radiographic, microtomographic, and histological analysis (n = 5). Radiographic analysis was carried out using a digital dental X-ray (Elytis, Trophy, MARNE LA VALLEE CEDEX 2, France at 60 kVp, 4 mA, film-focus distance of 20 cm and exposure time of 0.344 s). Radiographic analysis for micro-CT was carried out using X-ray tube voltage of 70 kVp with current intensity of 220 μA and integration time of 500 ms. Additionally, bone volume quantification was carried out using CTAn software (BRUKER, Billerica, MA, USA). For visual and display analysis, 3D images were also obtained. Histomorphometric analysis was carried out using Bioquant image analysis software (BIOQUANT Image Analysis Corporation, Nashville, TN, USA) to manually define the total bone area, newly formed bone area, and bone graft area.

### 2.7. Histological Evaluation

Fixed calvaria samples were processed for undecalcified histological preparation. Using EXAKT Grinding System (EXAKT Technologies, Norderstedt, Germany). Ground sections with 10–20 μm thickness were generated. All ground sections were applied with Masson–Goldner staining. Of the prepared ground sections, the most central section of the defect was selected. The central section was characterized by the ones displaying the widest extension. After the central sections of each defect area were identified, they were subjected to histologic and histomorphometric analysis. The defected area images are under 50× magnification and 200× magnification.

### 2.8. Statistical Analysis

Quantitative data calculation as means ± standard deviation and comparisons were carried out using one-way ANOVA (Systat Software Inc., Chicago, IL, USA). * *p* < 0.05 was considered statistically significant.

## 3. Results

### 3.1. Clinical Observation

During the 28 days post operation of the implant, no complications were observed from all groups. No significant pathological variations or presence of abnormal fluids were identified in any test specimens.

### 3.2. Characterization of Aln-Loaded Bone Graft

**Figure 3:**
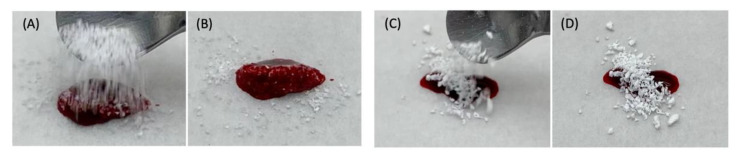
Comparison of wicking property between InRoad (**A**,**B**) and Bio-Oss (**C**,**D**) using fresh rat blood. Superior wicking property was demonstrated on InRoad compare to that of Bio-Oss.

There were no observed changes of the appearance and structure of both InRoad and Bio-Oss bone grafts associated with Aln loading. External morphology of both grafts was similar including porous cancellous structure (Figure 2A,B). Internal morphology of InRoad was clearly distinguishable compared to that of Bio-Oss with its micro-channel (#) structure inside trabecular septum, resulting in superior wicking property and enlarged surface area (Figure 2C,D). Lots of sub-micron-sized holes on the InRoad surface were observed to encourage cells to anchor (Figure 2E). Exceptional wicking property was demonstrated by dropping the bone graft onto fresh rat blood from both bone grafts (Figure 3). By dropping InRoad and Bio-Oss on the top of rat blood, InRoad was saturated with blood immediately without interruption (Figure 3A,B) while the Bio-Oss was floating on top of blood even after 7–10 min (Figure 3C,D).

The average loading amount of Aln on InRoad and Bio-Oss was 825.72 ± 8.03 μg and 547.60 ± 7.86 μg in Aln (1 mg), and 3601.05 ± 7.86 μg and 2752.35 ± 7.00 μg in Aln (5 mg), respectively. Their loading efficiency was 82.57 ± 0.80%, 54.76 ± 0.79%, 72.02 ± 0.16%, and 55.05 ± 0.14%, respectively (Table 1).

**Figure 4:**
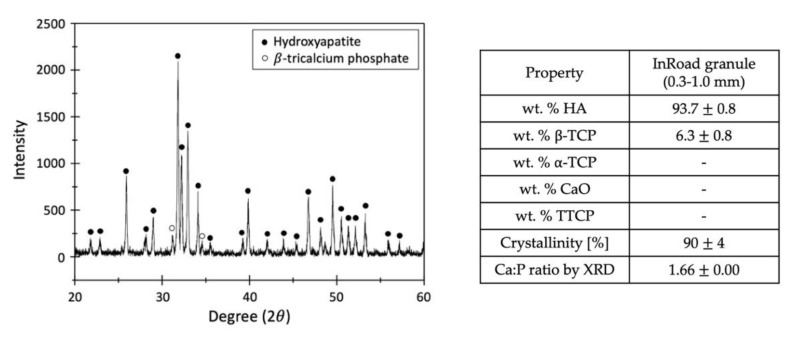
XRD for InRoad composed of 93.7% hydroxyapatite (HA) and 6.3% β-tricalcium phosphate (β-TCP).

InRoad (90 ± 5% HA and 10 ± 5% β-TCP) was synthesized by the reaction of calcium hydroxide and phosphoric acid. The powder diffraction files (PDF) used for analyses were 72-1243 (HA), 09-0169 (β-TCP), 09-0348 (α-TCP), 04-0777 (CaO), and 25-1137 (TTCP), in accordance with the International Centre for Diffraction Data (JCPDS-ICDD). Crystallinity was determined by calculating the percentage of pattern that was contained by any amorphous characteristic, such as the broad amorphous hump observed in the scraped samples. For the Ca:P ratio, the nominal peaks chosen for calibration were HA at 31.7° 2θ, α-TCP at 30.7° 2θ, β-TCP at 31.01° 2θ, and CaO at 37.4° 2θ. The ratio for Ca/P was 1.66, calculated by measuring their respective peak intensities using XRD pattern. The intense high and narrow crystallization peak are visible (Figure 4). The absence of decomposition phase, such as α-TCP or TTCP in HA and β-TCP, were confirmed.

### 3.3. Release Kinetic of Aln from Bone Grafts

**Figure 5:**
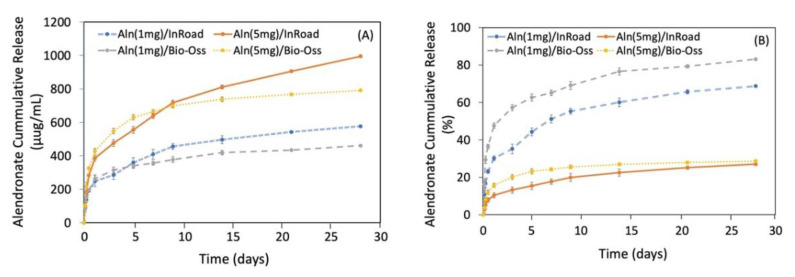
(**A**) Cumulative in vitro release profile of Aln from Aln (1 mg)/InRoad, Aln (1 mg)/Bio-Oss, Aln (5 mg)/InRoad, and Aln (5 mg)/Bio-Oss. Released Aln from both InRoad and Bio-Oss were similar within each concentration. (**B**) Percentage cumulative in vitro release profile of Aln showed that Aln concentration affected Aln releasing pattern. On the first day, 30.21 ± 1.09% and 47.62 ± 1.18% of Aln was released from Aln (1 mg)/InRoad and Aln (1 mg)/Bio-Oss, respectively. While 10.41 ± 0.99% and 15.71 ± 1.26% of Aln was released from Aln (5 mg)/InRoad and Aln (5 mg)/Bio-Oss, respectively. On the 28th day, 68.73 ± 1.15% and 83.09 ± 0.89% of Aln was released from Aln (1 mg)/InRoad and Aln (1 mg)/Bio-Oss, whereas 27.02 ± 0.67% and 28.70 ± 0.63% of Aln was released from Aln (5 mg)/InRoad and Aln (5 mg)/Bio-Oss.

Figure 5A showed the release profile of Aln from Aln (1 mg) and Aln (5 mg) on InRoad and Bio-Oss bone grafts. Sustained release of Aln up to 28 days was observed from all samples. On the first day, 248.54 ± 3.21 μg from Aln (1 mg)/InRoad, 263.29 ± 3.37 μg from Aln (1 mg)/Bio-Oss, 386.60 ± 3.39 μg from Aln (5 mg)/InRoad, and 429.02 ± 4.87 μg from Aln (5 mg)/Bio-Oss were released. A total of 577.27 ± 4.56 μg from Aln (1 mg)/InRoad, 461.40 ± 7.09 μg from Aln (1 mg)/Bio-Oss, 994.98 ± 3.38 μg from Aln (5 mg)/InRoad, and 791.38 ± 7.59 μg from Aln (5 mg)/Bio-Oss were released. The observed Aln release proportion differed depending on the Aln concentration for both InRoad and Bio-Oss. It was noted that higher concentration of Aln (5 mg) showed better sustension of Aln release. For 28 days, it was observed that 80% of Aln was released from Aln (1 mg)/Bio-Oss and less than 30% of Aln was released from Aln (5 mg)/Bio-Oss and Aln (5 mg)/InRoad. However, less than 70% (68.73 ± 1.15%) of Aln was released from Aln (1 mg)/InRoad (Figure 5B).

### 3.4. Analysis of Bone Formation

**Figure 6:**
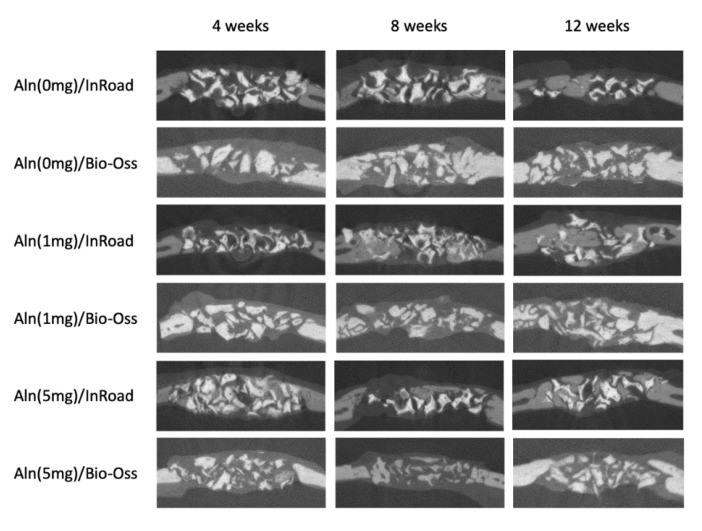
Micro-computed tomography (CT) analysis used to observe the amount of bone formation at the 4, 8, and 12 weeks after implantation. The amount of bone formation was observed using the calculated bone mineral density and bone formation volume (%BV). For the defect area, all tissues of the newly formed bone were included.

**Figure 7:**
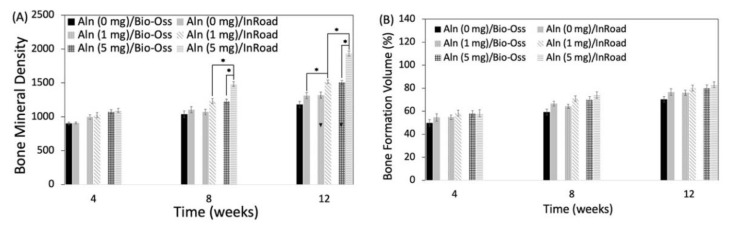
(**A**) Bone mineral density and (**B**) bone formation volume (%) at 4, 8, and 12 weeks after implantation. The error bars represent mean ± SD (n = 5). (* *p* < 0.05).

Using micro-CT, bone formation at 4, 8, and 12 weeks after implantation were evaluated. Figure 6 shows the disappearance of the sharp margin of implantation sites with a lapse of time. For some specimens, blunt margins were still observed until 8 weeks along with higher bone formation and radio-opaque consolidation of the defected area for both InRoad and Bio-Oss. Micro-CT images of InRoad specimens showed relatively consolidated new bone formation compared to Bio-Oss specimens throughout the study period, regardless of any concentrations of Aln. The bone mineral density and bone formation volume increased over time in an Aln-concentration dependent manner (Figure 7). Bone mineral density increased significantly at 8 and 12 weeks in Aln (5 mg) compared to that of Aln (1 mg) for both InRoad and Bio-Oss. Bone formation volume (%BV) was calculated as the percentage of new bone area in the surgical defect area. For the defect area, all tissues of the newly formed bone were included.

### 3.5. Histological Evaluation

Histology analysis at 50× and 200× magnification confirmed that Aln/InRoad bone grafts improved new bone formation. Masson–Goldner staining showed active new bone formation evidenced in all groups in an Aln-concentration-dependent manner. After 8 weeks of implantation, mature bone was distinctly observed around the residual materials in both InRoad and Bio-Oss in accordance with Aln release. After implantation for 12 weeks, the greatest extent of regenerated bone and blood vessels (arrowhead) were observed in Aln (5 mg)/InRoad group.

## 4. Discussion

Cell adhesion, proliferation, and mineralization are critical to be supported from bone graft materials in bone tissue engineering. Therefore, osteoconductive bone substitutes such as HA and β-TCP are usually considered. Although osteoconductive materials provide a framework for vascular and cellular infiltration, they lack the ability to stimulate the differentiation of mesenchymal stem cells or osteoblast-like cells. Therefore, synthetic bone grafts bring out more effective bone regeneration with the addition of osteoinductive materials such as Aln. The dominant role of Aln is to inhibit osteoclast function through the mevalonate pathway involving cholesterol synthesis inhibition [32]. However, recent studies revealed that local delivery of Aln using calcium phosphate scaffolds can also promote osteoblast differentiation and mineralization in vitro [33,34]. In addition, Aln is known to increase osteocalcin expression, mineralization, and unprenylated Rap1 in human mesenchymal stem cells [35]. Enhancement of proliferation and differentiation in bone-forming cells adjacent to the bone surface in vivo is also possible with the local treatment of Aln [36]. Local delivery of Aln can be explained as a two-step process, (1) preparing InRoad or Bio-Oss bone grafts and (2) the Aln loading on InRoad or Bio-Oss bone grafts. There are concerns of the limitations for the Aln loading process: heterogeneous distribution of Aln within the grafts and irregular release kinetics [37,38]. In this study, the characteristics of InRoad and Bio-Oss containing Aln were investigated to see whether Aln loaded InRoad or Bio-Oss enhanced new bone formation compared to that of Inroad or Bio-Oss without Aln using ovariectomize-induced osteoporotic rat calvaria defect model. In addition, by comparing the popular allograft with a synthetic bone graft, it was observed how much osteogenic property of the allograft could be supported with a synthetic bone graft.

The SEM images proved the porous structures even with the addition of Aln on both InRoad and Bio-Oss bone grafts. The InRoad bone graft is unique with its internal micro-channel structure with macro-pores and micro-holes on its surface. This unique physical characteristic provides larger surface area for cells to attach, encouraging cells to anchor and promote wicking property to enhance cells migration with blood (Figure 2). Simultaneously, this structural advantage is also beneficial for efficiency in Aln loading and releasing kinetics. In addition, macro-pores enable easy neovascularization and new bone ingrowth. Therefore, engineered micro-channels like that of InRoad demonstrates multiple functions. It provides double surface unlike any other bone grafts, resulting in a larger amount of Aln holding capacity and sustainable release capability, along with a larger space for new bone ingrowth. The larger space provided from InRoad achieving larger amount of new bone formation helps seamless integration between bone grafts and new bone after completion of defect healing. As shown in Figure 3, strong wicking property by the combinatorial structure of InRoad may have contributed to the active recruitment of heterogeneous cells from the host body with a surplus amount of blood. Essentially, without active cell migration from the surplus amount of blood in accordance with bone graft implantation, meaningful bone regeneration may be difficult. Hence, the wicking capability in any bone grafts is a crucial property for bone regeneration purposes. In this regard, InRoad may be considered as an innovative and beneficial approach to replace auto-, allo-, and xeno-grafts as a synthetic bone graft. The test results have been compared to the parameters specified by the standards and are summarized in Figure 4. InRoad granules exhibited Ca:P ratios that meet the ISO 13779 specifications.

A relatively linear Aln release kinetic was preserved over the 28-day period. A burst release was observed in the first 24 h (Figure 5A,B). It is hypothesized that the burst may have been due to the Aln bound on the surface of the bone grafts. The sustained and slower release of Aln from InRoad than that of Bio-Oss might be ascribed from the biphasic characteristics of InRoad (90 ± 5% HA and 10 ± 5% β-TCP) by dissolution of the calcium phosphate mineral [17,28,29,30]. Moreover, a naturally complicated structure like InRoad, composed of inner micro-channel structure and micro-holes, may consider the structural complication a favorable characteristic for better release kinetics than that of Bio-Oss. Considering the sustained release kinetics observed from this study, the Aln/InRoad bone graft may be capable of releasing Aln over several months. Therefore, Aln/InRoad bone graft shows a possibility to be used as a local long-term Aln delivery system for bone defect model in vivo.

**Figure 8:**
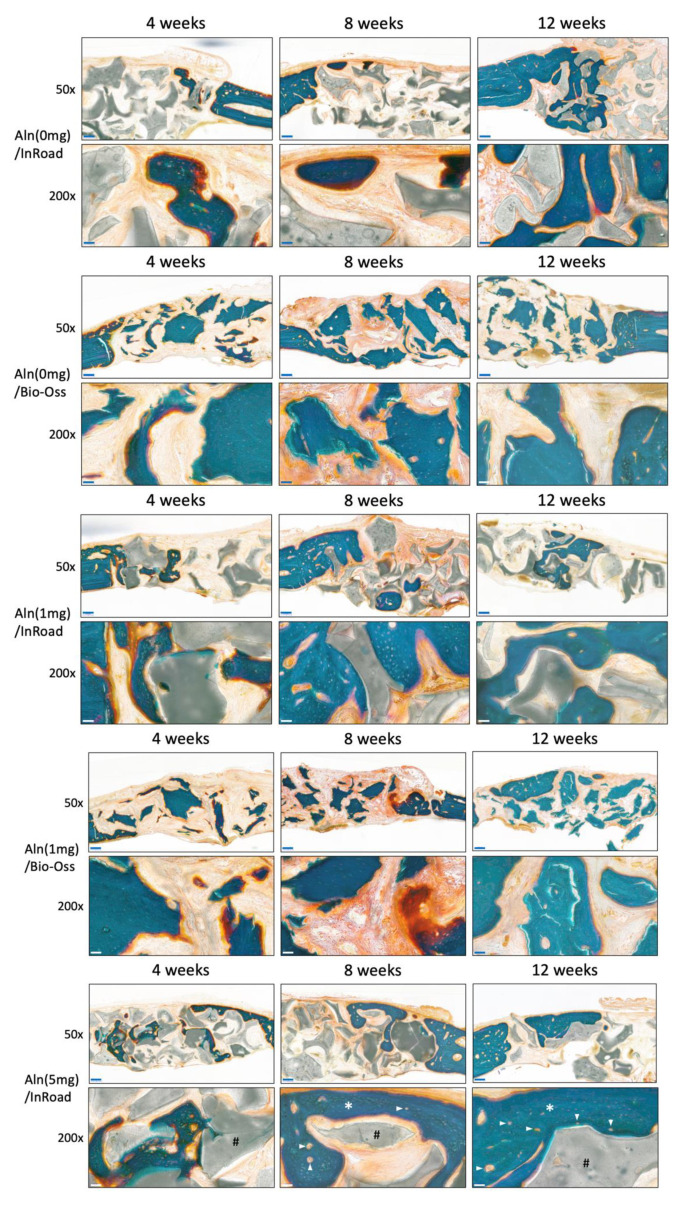

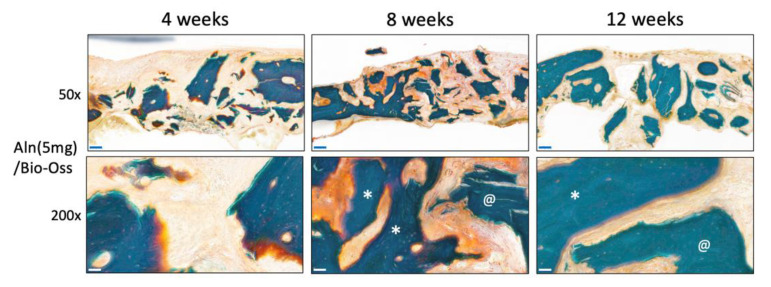
Representative sections of Masson–Goldner stains of 4, 8, and 12 weeks after implantation (50× magnification, 200 μm scale bar). Abundant surrounding fibrous tissue formation and woven bone formation were visible in the most of both samples, especially at 8- and 12-week point. Similar findings were observed on high magnification field (200× magnification, 50 μm scale bar). Blood vessels (arrowhead). InRoad bone graft (#). Bio-Oss bone graft (@). New bone formation (*).

The analysis from X-ray and micro-CT of Aln-loaded InRoad and Bio-Oss showed that bone growth was proportional to the amount of Aln content (Figure 6). No significance was observed when comparing bone mineral density and bone formation volume between 1 mg Aln loaded Bio-Oss and InRoad bone graft group and the control group (Aln-unloaded group) except at 12 weeks in InRoad group. However, significant differences in bone mineral density InRoad group at 8 and 12 weeks after implantation with increased Aln content (Figure 7) were observed. Whether increase in bone mineral density is due from the high doses of Aln or long-term exposure of Aln is difficult to determine. Through the histological analysis, it can be observed that Aln (5 mg)/InRoad bone graft showed woven bone formation at 4 weeks after implantation which most converted into mature bone at 8 and 12 weeks (Figure 8). These observations show Aln/InRoad bone graft has good potential for osteoinduction, osteogenesis enhancement, and bone regeneration. The mechanical strength of regenerated bone and patterns of biodegradation was unable to be determined.

Even with the well-known advantages of Aln, there are some concerns due to its uncoupling effect [39,40,41,42,43]. The over-suppression of bone turnover and osteogenesis inhibition are likely to be due to prolonged exposure and systemic administration of Aln [43,44]. All rats were observed up to 12 weeks after implantation and no adverse side effects were detected through the local Aln delivery through Bio-Oss and InRoad. Moreover, the Aln/InRoad bone grafts from this study showed a significant difference in bone formation over 12 weeks period with higher dose of Aln (5 mg) suggesting in vivo applications of Aln/InRoad synthetic bone graft (biphasic calcium phosphate: 90 ± 5% HA and 10 ± 5% β-TCP) is effective for bone regeneration.

There are limitations that are yet to be observed from the study. It is suggested to investigate the treated species for a longer period (more than 12 weeks after implantation) to observe the cumulative effect of Aln. With the elongated observation period, it may be possible to study how Aln affects the newly formed bone as well as the absorption of osteoclast-related material. Further evaluation with micro-CT is needed to test the length of bone consolidation, remodeling, and material absorption. In addition, biomechanical study of the regenerated bone is suggested for a deeper understanding of the structural stability and the quality of the regenerated area.

## 5. Conclusions

In this study, the bone regeneration feasibility and capability of granule type synthetic bone grafts with drug-carting ability were studied using osteoporotic rat model. The sustained release of Alendronate was demonstrated from both Aln/Bio-Oss and Aln/InRoad bone grafts. However, the slower and higher cumulating release of Aln was noticed from Aln/InRoad, along with enhanced bone formation and mineralization. The result was also in respect to the complicated macro–micro–submicron structure and superior wicking property of InRoad. In addition, InRoad synthetic bone grafts significantly enhanced the osteogenetic effect in an ovariectomize-induced osteoporotic rat calvaria defect model in vivo when compared with that of Bio-Oss. This presents great potential as a bone graft material with drug carrying ability for large bone defects even in osteoporotic clinical conditions.

## Figures and Tables

**Table 1:** Alendronate loading amount and efficiency in both InRoad and Bio-Oss in 1 mg and 5 mg concentration.

Samples	Loading Amount (μg)	Loading Efficiency (%)
Aln (1 mg)/InRoad	825.72 ± 8.03	82.57 ± 0.80
Aln (1 mg)/Bio-Oss	547.60 ± 7.86	54.76 ± 0.79
Aln (5 mg)/InRoad	3601.05 ± 7.86	72.02 ± 0.16
Ain (5 mg)/Bio-Oss	2752.35 ± 7.00	55.05 ± 0.14

## Data Availability

The data presented in this study are available on request from the corresponding author. The data are not publicly available due to intellectual property issues.
